# Application of causal inference methods in the analyses of randomised controlled trials: a systematic review

**DOI:** 10.1186/s13063-017-2381-x

**Published:** 2018-01-10

**Authors:** Ruth E. Farmer, Daphne Kounali, A. Sarah Walker, Jelena Savović, Alison Richards, Margaret T. May, Deborah Ford

**Affiliations:** 1 MRC Clinical Trials Unit at UCL, Institute of Clinical Trials and Methodology, UCL School of Life and Medical Sciences, London, UK; 2 Department of Non-communicable Diseases Epidemiology, London School of Hygiene & Tropical Medicine, London, UK; 3 Bristol Medical School, University of Bristol, Bristol, UK; 4 The National Institute for Health Research Collaboration for Leadership in Applied Health Research and Care West (NIHR CLAHRC West) at University Hospitals Bristol NHS Foundation Trust, Bristol, UK

**Keywords:** Causal inference, RCT, Systematic review, Time-dependent confounding, Marginal structural models, Marginal nested models, G-computation, G-estimation

## Abstract

**Background:**

Applications of causal inference methods to randomised controlled trial (RCT) data have usually focused on adjusting for compliance with the randomised intervention rather than on using RCT data to address other, non-randomised questions. In this paper we review use of causal inference methods to assess the impact of aspects of patient management other than the randomised intervention in RCTs.

**Methods:**

We identified papers that used causal inference methodology in RCT data from Medline, Premedline, Embase, Cochrane Library, and Web of Science from 1986 to September 2014, using a forward citation search of five seminal papers, and a keyword search. We did not include studies where inverse probability weighting was used solely to balance baseline characteristics, adjust for loss to follow-up or adjust for non-compliance to randomised treatment. Studies where the exposure could not be assigned were also excluded.

**Results:**

There were 25 papers identified. Nearly half the papers (11/25) estimated the causal effect of concomitant medication on outcome. The remainder were concerned with post-randomisation treatment regimens (sequential treatments, n =5 ), effects of treatment timing (*n* = 2) and treatment dosing or duration (*n* = 7). Examples were found in cardiovascular disease (*n* = 5), HIV (n = 7), cancer (*n* = 6), mental health (*n* = 4), paediatrics (*n* = 2) and transfusion medicine (*n* = 1). The most common method implemented was a marginal structural model with inverse probability of treatment weighting.

**Conclusions:**

Examples of studies which exploit RCT data to address non-randomised questions using causal inference methodology remain relatively limited, despite the growth in methodological development and increasing utilisation in observational studies. Further efforts may be needed to promote use of causal methods to address additional clinical questions within RCTs to maximise their value.

**Electronic supplementary material:**

The online version of this article (10.1186/s13063-017-2381-x) contains supplementary material, which is available to authorized users.

## Background

Well-powered randomised controlled trials (RCTs) are widely recognised to provide reliable and unbiased assessments of health interventions; however they require substantial effort and time, and are usually extremely expensive to conduct. Despite typically collecting large quantities of high-quality diverse data, for example, on laboratory parameters, concomitant medications and adverse events, the main focus of the RCT analysis is frequently a simple intention-to-treat (ITT) analysis of the randomised intervention.

For analyses other than those comparing randomised groups, RCT data are subject to the same issues of confounding and other potential biases as observational studies. It is generally well-known that in order to infer causal associations in such studies we must assume no unmeasured confounding; however, when the aim of the analysis is to examine the effect of a time-varying exposure, the issue becomes more complex. For example, we may be interested in examining the effect of antiretroviral therapy (ART) on survival in HIV-infected individuals. In this situation a patient’s CD4 count is a time-dependent confounder because it is a time-varying risk factor for survival, and it predicts when a subject is initiated on therapy. However, ART will also improve subsequent CD4 counts. When time-dependent confounders are affected by prior treatment, adjustment for the time-dependent confounder in a standard regression model will not appropriately adjust for the confounding.

The causal inference methods of g-computation [1], g-estimation [2], and, most commonly, inverse probability weighting (IPW) of marginal structural models (MSMs) [3] have been extensively applied in observational studies for dealing with time-dependent confounding [4–9]. However, their use in RCTs has predominantly focussed on adjusting for non-compliance with the randomised intervention [10, 11]. Briefly, as recently summarised by Naimi, Cole and Kennedy [12], g-computation models the joint distributions in the observed data to estimate potential outcomes under different exposure scenarios (and can be thought of as a longitudinal form of standardisation [13]). G-estimation relies on the assumption of no unmeasured confounding to estimate the parameters of a set of structural nested models, in which the effect of the exposure is broken down incrementally. IPW of MSMs re-weights the population so that the exposure becomes independent of time-varying confounders.

The scope of causal methodology is broad and could be used to exploit clinical trial data to address many questions beyond analyses of the randomised intervention. For example, existing methodology could allow questions about effectiveness of concomitant medications, treatment switching and optimal dynamic treatment strategies (where treatment is altered in response to patient characteristics that change through time) to be examined, which would add significant value to the output of a single RCT.

In this review we aimed to identify published studies exploiting causal inference methodology to deal with time-dependent confounding, which used clinical trial data to examine questions that were not addressed by the trial randomisation. In doing this, we aimed to gain an overview of how widely such methodology is used in the clinical trial context, and identify examples of the value gained through use of these methods.

## Methods

We aimed to identify all studies in any clinical area that exploited causal inference methodology using clinical trial data. To achieve this we used both a keyword search in Medline, Premedline, Embase, Cochrane Library and Web of Science, from 1986 to September 2014; and a forward citation search of five seminal papers [1, 3, 9, 14, 15].

### Search strategy

We worked with an information specialist/research librarian and a systematic reviewer to develop the search protocol and our information specialist undertook the primary search. The details of the protocol are documented in Additional file 1: Appendix 1. Keywords were identified by the authors and the information specialist, and the searches were set up by the information specialist using a combination of index headings (where available) and text word searching. In simplified form, the keyword search included the following terms: ((time-varying confounding OR causal effect or parameter OR causal inference) AND (marginal structural models OR inverse probability weighting OR g-estimation OR g-formula OR structural nested models)) OR one of the five key citations. Full details of the searches undertaken and their results are provided in four parts corresponding to the Medline, Cochrane, Embase and Web of Science biomedical databases, respectively in Additional file 1: Appendix 2. Searches were conducted on 5 September 2014 and were limited to English language material, excluding animal studies, case reports, letters, editorials and economic analyses.

### Screening

Papers identified by the search strategy were initially screened for eligibility by one author using Covidence systematic review software, Veritas Health Innovation, Melbourne, Australia. Available at (www.covidence.org). The initial eligibility criteria, based on an abstract screen, were as follows: (1) use of any of the causal methods defined in the search and (2) use of clinical trial data. If studies appeared to fulfil those criteria they were obtained in full text and reviewed for inclusion using a priori exclusion criteria as follows:

1. Causal method not used
2. Theory only with no application to either real or simulated data
3. Conference abstracts with no information to allow assessment of methods
4. Tutorial pieces or observational studies
5. Applications using IPW only to address baseline imbalances for the comparisons of interest or informative loss to follow up (or both)
6. Applications using causal inference methods only to address non-adherence to randomised treatment, unless the issue of non-compliance was a question of dosage or duration of treatment and causal inference methods were used to infer information about the optimal dosage/duration
7. Studies of exposures that cannot be assigned e.g. lifestyle exposures such as body mass index (BMI), exercise or socio-economic deprivation
8. Theory papers with simulation only
9. Theory papers where the application was in observational data (if not picked up by exclusion criterion 4)

During review we identified a small number of papers that described analysis of a sequential multiple assignment randomised trial (SMART) where IPW based on randomisation was used to estimate outcomes under embedded adaptive interventions; these studies were ineligible based on criterion 5, but we chose to create an additional 10th exclusion criterion:

10. Analysis of SMART designs

The full-text screen to establish exclusion based on criteria 1–4 was performed by a single author, with the remaining studies reviewed independently by four authors against criteria 5–10. Any discrepancies were resolved via a group discussion.

### Data extraction and categorisation of the causal question

Data extraction was performed in duplicate by REF and DK. Following the review aims, the key information extracted from each study included details of the original trial (including an overview of the trial population, details of the randomised comparison); the causal question of interest and any refinement of the study population in order to answer this question; the causal method used and the key references given and both the trial result and the results of the causal analysis. The full completed extraction table is available in Additional file 2.

Each paper identified was categorised into one of 4 types of causal question developed during the data extraction phase. This was done to describe the kind of questions that were already commonly looked at, and also to identify those less frequently examined, but with potential to be more widely applied to other situations in the future. The question types identified were as follows:

1. Concomitant medication – this covered all studies looking at the effect of any additional non-randomised medications or treatments that were used during the trial period
2. Sequential treatments – encompassing studies examining the effect of different post-randomisation treatment regimens, or comparison of/adjustment for second-line treatments, which were dependent on response to first-line treatment.
3. Treatment timing – including all studies that looked at the timing of second-line or post-randomisation treatments
4. Treatment dosing/duration – studies that examined the effect of non-randomised dosing strategies or duration of treatment.

## Results

**Fig. 1:**
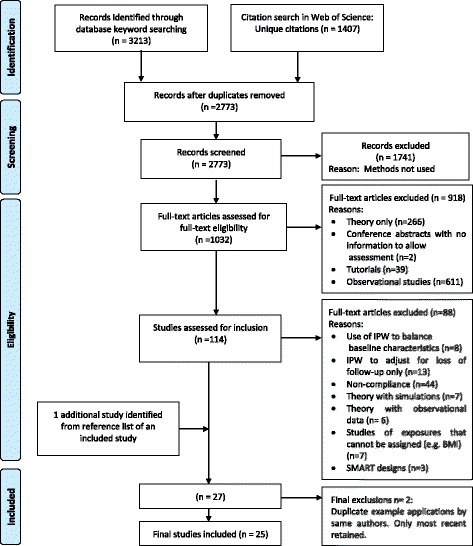
Preferred reporting items for systematic reviews and meta-analyses (PRISMA) flow chart. *IPW* inverse probability weighting, *SMART* sequential multiple assignment randomised trial

From a total of 2773 studies retrieved (after removing duplicates) from the search, 1032 were initially screened for having potential relevance. From these, 114 were identified for detailed full-text screening, and 26 papers satisfied all inclusion criteria. The process of study identification, screening and inclusion is summarised in the preferred reporting items for systematic reviews and meta-analyses (PRISMA) flowchart (Fig. 1) [16]. The PRISMA checklist corresponding to the review is presented in Additional file 3.

Two of the 26 studies were found to be similar applications to the same data by the same authors as two other included studies. To avoid replication, only the most recent publication for each pair was taken forward for data extraction, with the earlier publication noted in the extraction table. Additionally, at the data extraction point, an additional relevant study was identified from the reference list of an included study, and was added. Therefore the final number of included studies was 25.

The papers covered six broad research areas: seven studies in HIV, six in cancer, five in cardiovascular disease (two any cardiovascular disease, three diabetes), four in mental disorders, two in paediatrics and one in transfusion medicine. The majority of papers (n = 11) estimated the causal effect of concomitant medication: 7 looked at treatment dosing/duration, 5 at sequential treatments, and 2 treatment timing. Table 1 provides a brief summary of each study, with details of the original trial question, the causal question examined, the method used and findings.

**Table 1:** Summary of included studies, including the disease area, original trial question, category of causal question, methods used and result of causal analysis

Study	Disease area	Trial question	Causal category	Causal question	Causal method	Causal result summary
Alexander (2014) [22]	Cardiovascular disease	Comparative effectiveness of apixaban vs warfarin in patients with atrial fibrillation for risk of cardiovascular and thromboembolic endpoints	Concomitant medication	What is the effect of aspirin use on risk of cardiovascular and thromboembolic endpoints?	MSM with IPW [9]	Evidence of an increased risk of stroke or major bleeding from aspirin use as a concomitant medication on top of apixiban or warfarin
Bobo (2014) [26]	Mental health	Comparative effectiveness of lithium vs quetiapine on clinical outcomes in patients with bipolar disorder	Concomitant medication	What is the effect of benzodiazepine use on core bipolar mood symptoms?	MSM with IPW [3]	No statistically significant effect of benzodiazepine use on any of the outcomes, though there were extremely wide confidence intervals both before and after IPW
Cook (2013) [43]	Transfusion medicine	Non inferiority of pathogen inactivated platelets vs standard platelets for successful blood transfusion (MIRASOL study)	Dosing/duration	What is the probability of overall success in increasing platelet count over 28 days while correctly taking into account number of transfusions needed?	MSM with IPW [3, 6, 9]	A reduced probability of transfusion success with increasing transfusion number was observed in the weighted analyses, but the effect was smaller than was observed in the original trial analysis
Crook (2014) [17]	HIV	Safety and efficacy of a microbicide gel vs placebo to prevent HIV transmission in women with HIV positive partners in Sub Saharan Africa (MDP301 trial)	Concomitant medication	What is the causal effect of hormonal contraception (injectable and oral) on HIV incidence?	MSM with IPW [3, 14]	Evidence of an elevated risk of HIV infection with use of DMPA but no other hormonal contraceptives.
Kataoka (2012) [23]	Diabetes	Efficacy of voglibose, nateglinide and lifestyle interventions on changes in coronary atherosclerosis in patients with early-stage diabetes mellitus	Concomitant medication	What is the effect of treatment with statins, ACE inhibitors and ARBs (considered separately as three classes of concomitant medications) on atheroma progression as measured by total lesion length (TLL)?	MSM with IPW [3, 9]	Use of statins was estimated to increase TLL. No evidence of an effect of any other concomitant medications examined
Li (2012) [36]	HIV	Placebo-controlled comparison of three ART regimens: ABC/3TC/ZDV, 3TC/ZDV + EFV, ABC/3TC/ZDV + EFV	Treatment timing	What is the effect of switching early (within 8 weeks) vs late (after 8 weeks) onto a second-line therapy after first virologic failure on CD4 count and other clinical markers of HIV severity?	IPW [37]	Switching early results in better clinical outcomes for all three clinical measures examined than switching late
Lipkovich (2008) [41]	Mental health	Trial 1. Efficacy and safety of olanzapine vs haloperidol in terms of clinical scores and quality of life in acute bipolar patients Trial 2. Efficacy and safety of olanzapine vs risperidone in terms of clinical scores in schizophrenic patients	Dosing/duration	What is the effect of dosage of olanzapine on change in disease severity measures? Estimated separately in each trial	MSM with IPW [3, 9, 49]	No strong evidence of differences in response with changing dose of olanzapine, though in one study the weighted analysis was suggestive of a negative dose effect at weeks 20 and 24 only.
London (2010) [34]	Cancer	Superiority of TOPO vs TOPO with CTX (chemotherapy combinations) for response to treatment in recurrent or refractory neuroblastoma in children.	Sequential treatments	What is the best chemotherapy combination for first line treatment after adjusting for the optimal off-protocol treatment for survival at 2 years?	G estimation of an optimal Structural Nested Model [29, 35]	Under the assumption that all patients received the optimal off-protocol therapy, there were no detectable differences between TOPO and TOPO with CTX (2-year survival 40% vs 33%, *p* = 0.215)
McCoy (2013) [19]	HIV	Safety and efficacy of diaphragm, lubricant gel and condoms vs condoms alone to prevent HIV transmission in women in Sub Saharan Africa (MIRA study)	Concomitant medication	What is the effect of hormonal contraception (injectable and oral) on HIV incidence?	MSM with IPW [51]	No strong evidence of effect of oral contraceptives on HIV transmission, small suggestion of increased risk for injectable contraception
Morrison (2012) [18]	HIV	Safety and efficacy of a microbicide gel vs placebo to prevent HIV transmission in women in South Africa (Carraguard study)	Concomitant medication	What is the effect of hormonal contraception (injectable and oral) on HIV incidence?	MSM with IPW [3, 6]	No strong evidence of effect of oral or injectable contraceptives on HIV transmission, but possibility of an increased risk with use of DMPA
Rosenblum (2009) [20]	HIV	Safety and efficacy of diaphragm, lubricant gel and condoms vs condoms alone to prevent HIV transmission in women in Sub Saharan Africa (MIRA study)	Concomitant medication	What is the effect of the intervention controlling for condom use as a mediator, and what is the direct effect of condom use on HIV transmission?	MSM with IPW [6]	No evidence of a difference in HIV risk between treatment arms after controlling for overall condom use. Condom use was estimated to be protective in both trial arms, though was only statistically significant in one arm
Rosthøj (2011) [39]	Cancer	Effect of blood counts only (control) vs blood counts and pharmacokinetic parameters to make therapy decisions on relapse in acute lymphoblastic leukaemia in children.	Dosing/duration	How should dose of oral therapy be adjusted (increase, no change, decrease, pause) in response to a given white blood cell count to obtain a future white blood cell count within the target range within a given time frame (e.g. within two weeks). How do the estimated optimal strategies compare to the original protocol?	History Adjusted MSM using IPW [40]	Optimal strategy estimated by the model was broadly consistent with the actual protocol for treatment dosing; however, where protocol suggested moderate reduction in dose, the MSM more often suggested no change, and where a moderate increase in dose was suggested by protocol, the MSM more frequently suggested a large increase to be the optimal choice.
Severus (2010) [42]	Mental health	Efficacy of olanzapine vs lithium in prevention of mood episode relapse or recurrence in patients with bipolar disorder	Dosing/duration	What is the effect of dosage of lithium and olanzapine on recurrence of symptoms?	MSM with IPW for probability of high/low dose at each interval [3]	High and medium lithium doses were shown to reduce risk of manic/mixed episode compared to low doses, but here was no evidence of an effect on risk of depressive episodes as confidence intervals were wider (although estimates were similar) Higher doses of olanzapine were associated with lower risk of depressive episodes but not manic/mixed episodes
Shen (2013) [24] (similar analysis also presented in [52])	Diabetes	Efficacy of two drugs (nateglinine or valsartan) on conversion to diabetes and CV outcomes in patients with impaired glucose tolerance and other CV risk factors.	Concomitant medication	What is the effect of use of beta blockers, diuretics, and statins on onset of Type 2 diabetes?	MSM with IPW [3]	Use of statins and diuretics found to increase risk of diabetes onset
Shinozaki (2012) [27]	Diabetes	Effect of intensive (target HbA1c 6.9 or less, BMI < 25, BP < 130/80) vs conventional treatment (no target levels) strategies for type 2 diabetes in elderly patients on risk of morality, cardiovascular events and other diabetes related endpoints	Concomitant medication	Is there a preventative effect of atorvastatin on cardiovascular disease and on diabetic vascular complications?	MSM with IPW, structural nested failure time models with g-estimation [3, 53]	Atorvastatin estimated to be protective against both cardiovascular events and diabetes related events, though confidence intervals for cardiovascular events were very wide.
Shortreed (2012) [38]	Mental health	Efficacy and tolerability of antipsychotic medications (perphenazine vs olanzapine/risperidone/quetiapine/ziprasidone) in patients with schizophrenia on time to failure of treatment (time to first switch)	Treatment timing	What is the optimal therapeutic strategy in terms of initial threshold of PANSS score (a scale of psychotic symptoms) to switch to an atypical antipsychotic drug over perphenazine to minimise schizophrenic symptoms and maximise quality of life over 12 months?	Dynamic MSM with IPW [54]	No differences detected between the different dynamic regimes for 12-onth quality of life. All strategies predicted improvements in PANSS by 12 months. There was no significant difference between 12 months PANSS score between the strategies of “always treat with perphenazine” and “always treat with atypical antipsychotic”, but it was observed that if starting with perphenazine, PANSS at 12 months was increased if the threshold to switch was higher (i.e. when the patient was more likely to switch).
VandeboscH (2005) [44]	HIV	Safety and efficacy of a microbicide gel vs placebo to prevent HIV transmission in women in Africa	Dosing/duration	What is the effect of cumulative gel use on development of vaginal lesions prior to HIV diagnosis?	Structural accelerated failure time model [55]	Increased experimental gel use was found to reduce time to first or any lesion compared to placebo gel, suggesting it may increase risk of lesions compared to placebo gels.
Wahed (2013) [31]	Cancer	Effect of adding all trans retinoic acid (ATRA), granulocyte colony stimulating factor (GCSF) or both to fludarabine plus cytosine arabinoside plus idarubicin (FAI) as a first-line (induction) therapy on the probability of success (alive and in complete remission at 6 months) in treatment of acute leukaemia	Sequential treatments	What is the best combined induction (randomised) and salvage (non-randomised) strategy to improve overall survival?	G formula MSM with IPW [1, 3, 56]	Both methods gave results consistent with FAI plus ATRA being the best remission induction therapy. If patient’s disease was resistant to that therapy, then salvage therapy options were equivocal. If a patient relapsed after CR with FAI plus ATRA then salvage therapy with HDAC was superior
Walker (2010) [21]	HIV	Effect of laboratory monitoring in addition to clinical monitoring of patients with HIV in Africa on clinical endpoints and mortality.	Concomitant medication	What is the effect of use of cotrimoxazole on survival, WHO stage 3 and 4 events, malaria and CD4 counts in adults after ART initiation?	MSM with IPW [3]	Use of cotrimoxazole was estimated to improve survival and reduce malaria infection in adults on ART Reduction in mortality was greatest in first 18 months of ART
Wang (2012) [28]	Cancer	A sequential multiple assignment randomised trial (SMART) designed to evaluate and compare 12 different sequential rules for switching from initial combination chemotherapy to second chemotherapy in prostate cancer, in terms of overall response to treatment and overall survival.	Sequential treatments	What is the best overall strategy to maximise response to treatment (“success”) after re-defining the possible treatment regimens to be viable treatment regimens allowing switches to non-randomised therapy.	Dynamic MSM with IPW [30, 37]	Different combinations were estimated to be optimal based on different measures of “success” Wide confidence intervals and large number of comparisons meant statistical comparison between actual scores was not feasible
Zhang (2012) [25]	Cardiovascular disease	Effect of felodipine in addition to hydrochlorothiazide after 6 weeks of therapy (i.e. intensive blood pressure reduction vs standard care) in a population of Chinese patients at risk of cardiovascular disease, on reduction in various cardiovascular endpoints (e.g. stroke)	Concomitant medication	What is the effect of add-on therapy (in the form of an increased dose of hydrochlorothiazide and/or other antihypertensive agents excluding calcium antagonists) in addition to randomised therapy on trial outcomes?	MSM with IPW [9]	Absence of add-on therapy was found to be protective for stroke, all-cause mortality and all cardiovascular events, despite the add on therapy reducing systolic and diastolic blood pressure
Zhang (2013) [33] (similar analysis also presented in [57])	Cancer	Effect of petrexemed in addition to cisplatin in patients with malignant pleural mesothelioma in terms of time to response and overall survival	Sequential treatments	What is the effect of the randomised first-line treatment if secondary treatments and discontinuation of study treatment are correctly adjusted for?	MSM with IPW [6]	Addition of petrexemed found to be beneficial in comparison to cisplatin only, with a smaller HR estimated via causal methods compared to ITT analysis
Yamaguchi (2004) [32]	Cancer	Superiority of CPT-P vs VDS-P and non-inferiority of CPT vs VDS-P (chemotherapy combinations) for survival in non-small cell lung cancer	Sequential treatments	What is the direct effect of the randomised treatment for the (CPT-P vs VDS-P) on survival, accounting for imbalances in second-line therapy?	MSM with IPW, G –estimation of structural nested models [3, 49, 58]	CPT-P was not shown to be beneficial compared to VDS-P and no difference was observed between the two different second line therapies in terms of survival
Platt (2012) [45]	Paediatrics	Effect of breast feeding intervention on prevalence and length of exclusive breastfeeding, and weight, height, and infection rates by 12 months	Dosing/duration	What is the effect of length of breast feeding on weight (kg) at 12 months?	MSM with IPW [3]	Fitted mean 12-month weight (kg) highest for 2 months exclusive breastfeeding and lowest for 9–12 months exclusive breastfeeding
Moodie (2009) [46]	Paediatrics	Effect of breast feeding intervention on various 12-month endpoints such as prevalence and length of exclusive breastfeeding; and weight, height, and infection rates in children	Dosing/duration	What is the effect of length of breast feeding on weight (kg) and length (cm) at 12 months?	G-estimation of structural nested models [29, 35, 59]	Breastfeeding to at least 9 months optimised 12-month weight and length

*MSM* marginal structural model, *IPW* inverse probability (of treatment) weighting, *DMPA* depo-medroxyprogesterone acetate, *ART* antiretroviral therapy, *TOPO* topotecan, *CTX* cyclophosphamide, *ABC* abacavir, *3TC* lamivudine, *ZDV* zidovudine, *EFV* efavirenz, *CPT-P* irinotecan plus cisplatin, *VDS-P* vindesine plus cisplatin, *CPT* irinotecan, *ACE* angiotensin-converting enzyme, *ARBs* angiotensin II receptor blockers, *PANSS* positive and negative syndrome scale, *WHO* World Health Organisation, *HR* hazard ratio, *ITT* intention to treat

### Concomitant medication

Of the 11 studies that examined questions about concomitant medication, 5 were in cardiovascular disease, 5 in HIV, and 1 in mental health: 4 HIV studies were based on trials designed to examine efficacy of microbicides for preventing HIV infection in HIV-seronegative women in Sub-Saharan Africa (MDP301 [17], Carraguard [18] and MIRA [19, 20]). The causal question of interest in three of these studies was the effect of hormonal contraceptives (oral and injectable) on acquisition of HIV infection, with appropriate control for time-dependent confounders. All studies used some form of IPW of MSMs to do this. All three studies found similar results, in that there was no evidence of an effect of oral hormonal contraception use on HIV incidence, with some suggestion of an increased risk with the injectable contraception depo-medroxyprogesterone acetate (DMPA). Although some of the estimates changed slightly, the causal methods produced broadly similar results to standard analysis methods in these cases. The fourth study [20] aimed to look at the effect of the microbicide controlling for condom use as a mediator, and also to estimate the effect of condom use itself. The final study in HIV, which also applied IPW, demonstrated a benefit for concomitant use of cotrimoxazole (an antibiotic) in patients starting ART in Africa, on mortality and malaria [21].

Data from much larger trials were available in the area of cardiovascular disease. For example the ARISTOTLE [22] international mega-trial was designed to assess the efficacy and safety of apixabin versus warfarin in patients with atrial fribrilation (AF). The causal inference analysis aimed to establish the effect of concomitant use of aspirin, which was prescribed at the discretion of the treating physician in addition to the randomised treatment. As with the majority of papers examining questions relating to concomitant medication, the method implemented was a marginal structural model (MSM) with IPW. In this case, the IPW estimates indicated that the risks of stroke and major bleeding with aspirin use were underestimated when standard analysis was performed, increasing the hazard ratio (HR) for stroke from 1.18 (0.94–1.49) to 1.46 (1.15–1.85) and for major bleeding from 1.41 (1.21–1.66) to 1.65 (1.40–1.94). Three of the other studies in cardiovascular disease [23–25] and the mental health application [26] also used MSMs with IPW. Finally, a study by Sinozaki et al. [27] investigated the effect of atorvostatin on various cardiovascular outcomes (including low-density lipoprotein (LDL) cholesterol, composite cardiovascular event endpoints, diabetes-related endpoints) by using both MSMs with IPW and g-estimation of structural nested models. The authors found that both methods produced relatively similar results for all outcomes examined.

### Sequential treatments

Analyses to compare treatment sequences were all from cancer trials. Such trials commonly involve treatment switches and second-line therapies that depend upon the patient’s response to the randomised first-line treatment. Causal inference analyses are then necessary in order to establish the optimal combination of treatments. For example, Wang et al. [28] used data from a sequential, multiple assignment, randomised trial (SMART) in advanced prostate cancer to demonstrate the use of dynamic marginal structural models (dMSMs) with IPW [29, 30] to estimate the overall optimal strategy to maximise response to treatment. This analysis was different to those originally conducted and reported for the trial, because the original analysis did not appropriately account for patients experiencing severe toxicity or disease progression (at which point non-randomised treatment decisions were made). A second application used both g-computation and dMSMs to examine the relative success of different combinations of induction (first) and salvage (second) treatments for acute leukaemia [31].

Yamaguchi et al. [32] used both a structural nested model (SNM) and an MSM with IPW to adjust for receiving a secondary treatment for non-small-cell lung cancer. This analysis, rather than identifying an optimal strategy including different secondary treatment options, estimated the effect of the randomised comparison under the assumption that everyone received the same secondary treatment, and additionally looked at the direct effect of secondary treatment on survival. A similar question was addressed via the use of MSM with IPW by Zhang and Wang [33] in the context of treatment for malignant pleural mesothelioma, and by London et al. [34], who used g-estimation of SNMs [35] to compare 2-year survival rates of two first-line treatment strategies for children with neuroblastoma, while adjusting for the optimal off-protocol therapy.

### Timing of treatment

Two studies examined questions relating to timing of treatment. Li [36] used data from a trial comparing three ART regimens in HIV-infected adults to look at whether early vs late treatment switch after first virologic failure had an effect on future viral load and CD4 cell count. The authors perfomed an analysis based on the theoretical 2001 paper by Murphy, van der Laan and Robins [37], which presented an IPW estimator for the comparison of dynamic strategies, and found that early switch after failure was beneficial compared to late switch. This was in contrast to an unweighted analysis restricted to patients who experienced virologic failure, which showed no evidence of a difference between strategies.

The second example was in the area of mental health. The Clinical Antipsychotic Trials of Intervention Effectiveness (CATIE) trial was a large multistage trial aiming to assess the long-term efficacy and tolerability of newer atypical antipsychotics compared to standard antipsychotics in the management of schizophrenia. The protocol allowed for treatment switching in response to success of the initial treatment. Shortreed et al. [38] used a subsample of the trial population to estimate the effect of different switching thresholds on minimizing schizophrenic symptoms at 12 months, by employing dMSMs with IPW. The authors found no evidence of differences between always treating with atypical vs standard antipsychotics, but that it was more beneficial to remain on the standard antipsychotic than switch, no matter what the observed response to therapy.

### Treatment dosing

The Nordic Society for Pediatric Hematology and Oncology Acute Lymphyblastic Leukaemia (NOPHO-ALL-92) was a trial in children with ALL, treated long-term with intensive chemotherapy. It was designed to assess a new treatment strategy, where at the maintenance phase patients received oral doses of drugs tailored to their blood counts, which were monitored weekly. Rosthøj [39] presents an application of history-adjusted MSMs [40] to data from this trial, whereby the estimated optimal dosing strategies were examined and compared to those set out by the protocol. In general, the optimal strategy estimated by the model was broadly consistent with the actual protocol for treatment dosing. However, where the protocol suggested a moderate reduction in dose, the MSM more often suggested no change, and where a moderate increase in dose was suggested by protocol, the MSM more frequently suggested a large increase to be the optimal choice.

In mental health studies, patients not only switch between different drugs but also receive dose-adjustments, which are often at the clinician’s discretion, even in clinical trial settings. Such dose adjustments through time will depend on many factors, which have likely been influenced by prior dosing decisions – a typical example of time-dependent confounding affected by prior treatment. Two studies [41, 42] used data from three flexible dose trials in acutely ill patients with bipolar disorder and schizophrenia. Both fitted MSMs with IPW to correctly adjust for confounding.

Another application of IPW to investigate dosing was found in transfusion medicine with repeated binary outcomes [43]. In the MIRASOL study, two platelet types were compared for non-inferiority in terms of overall successful transfusion for 28 days after surgery, where multiple transfusions could be performed. However, the effect of transfusion number on the probability of success of the transfusion (which was an intended secondary analysis of the trial) could not be correctly estimated without the use of causal methodology since those not responding to their first transfusions were more likely to need more, resulting in an estimated negative effect of multiple transfusions. The authors showed that this effect was attenuated by the use of causal methods.

An analysis in HIV infection, again based on data from a trial of a microbicidal gel, examined the effect of “dosing” by examining cumulative exposure to the experimental gel in relation to development of lesions [44]. This study was motivated by the original trial finding that the experimental gel actually increased HIV transmission. To see if the reason for such a finding was due to the gel causing lesions, causal methods were necessary to estimate the effect of cumulative gel use on lesion development, with appropriate control for the number of sexual acts, which itself likely influences lesion development. The authors used structural, accelerated failure-time models extended to deal with multiple events and found that the survival time to both first, and all lesions, was shorter in the experimental arm than the placebo arm, and that the relative difference in survival time between arms increased as gel dose increased.

Finally, two studies used data from the same cluster randomised trial of a breastfeeding intervention to look at the effect of duration of breast feeding on infant weight and length at 12 months. To adjust for confounders of the association between length of breastfeeding and infant weight and length, one study used MSM with IPW [45], and the other g-estimation of SNMs [46]. In a previous publication, a non-causal analysis had estimated that both weight-for-age and length-for-age were higher in the first 3 months in babies breastfed for more than 3 months and more than 6 months, respectively, compared to those weaned before 1 month. From 6 months onwards, longer exposure to breastfeeding appeared to reduce weight-for-age and length-for-age compared to early weaning. The MSM estimated that mean weight at 12 months was highest for children exclusively breastfed to 2 months. The expected 12-month weight was observed to reduce as the length of breastfeeding increased to 9 months. The estimated weight at 12 months was the same for 9 or 12 months duration of breastfeeding. In contrast, using SNMs it was found that continuing to 9 months would increase weight and length, but that additional breastfeeding beyond 9 months would not increase 12-month weight or length further. The authors of [46] discuss potential reasons for the difference in results between the two causal methods, with one possible explanation being the way the two methods handle subjects with missing data.

## Discussion

There are applications of causal methodology to data from RCTs across a number of disease areas and research questions, though the number of such applications is fairly low. The most commonly addressed question type is the effect of concomitant medication on outcome, with about half the papers studying this. This is likely because it is a common question of interest when examining clinical trial data; particularly, for trials in chronic disease populations, it is unlikely that the randomised treatment will be the only therapy being taken for disease management or to control other comorbidities throughout follow-up. Second, it is a question that can be easily addressed, given enough prescribing variability, through the use of MSM with IPW, which, with its link to sampling weights and propensity score weighting, is perhaps the most intuitive and easily implemented of the causal methods.

The use of causal methods to look at dosing (seven studies), and sequential treatments (six studies) was less common. It is possible that this is due to a lack of variability in dosing or second-line treatment options in the specified trial protocols, or because fewer data are collected if a patient deviates from the trial protocol. The sequential treatments question was most common in cancer trials, likely due to this being a disease in which interest lies in the success of a complete treatment strategy rather than the direct effectiveness of individual treatments.

Questions relating to timing of treatment were not frequently examined. Applications were limited to one paper in HIV, which looked at early versus late treatment switch after the first virologic failure, and one paper in mental health, which compared switching strategies based on a single measure of treatment performance. Other timing questions that could also be investigated via the use of the same methodology could be related to the level of laboratory or clinical measures at which treatment should be initiated rather than intensified/switched, or could be extended to compare more complex switching rules containing multiple variables. For example, subsequently to this review Ford et al. [47] used dynamic MSMs to investigate optimal treatment strategies for switching patients on ART including when to define failure (based on CD4 threshold or clinical event history) and how frequently to monitor CD4 count.

The ability to model these dynamic strategies relies on observing multiple strategies within the study data. In many settings, trials allow for non-randomised secondary treatments if the randomised treatment is considered to have failed, enabling researchers to investigate sequencing questions provided the necessary data are collected after initial failure. However, if the threshold for “failure” of treatment is clearly specified in the protocol, there may not be enough variation in the observed treatment strategies to examine questions of treatment timing. Noting the exception already described above [47], it may be the case that, for questions of treatment timing, causal analysis of observational data (in which greater variation in levels of key measures that define “failure” at the time of treatment switching will be observed) are more useful for examining questions of treatment timing and generating hypotheses for subsequent trials of treatment strategies.

Of the 25 papers identified, 12 came from medical journals. Of these, 10 were questions on concomitant medication using IPW of MSMs. It therefore appears that the current literature, particularly for questions other than those on the effects of non-randomised treatments, remains focussed towards those interested in statistical methodology. It may be that those focussed towards clinical research and trials may still lack awareness of the types of alternative questions that can be answered, and of alternatives to IPW of MSMs; or that the methodology is still limited in terms of its ability to draw strong clinical conclusions and is therefore of less interest to medical journals. Within cancer, the literature highlights the need for causal methodology. For example, an article in the Journal of Thoracic Disease [48] discusses the problem of time-varying confounding in clinical trials exploring the long-term effects of first-line treatment in patients with cancer and warns about the lack of analyses implementing causal methodology to adequately and appropriately assess treatment effects. One research area in which causal methods seem more established is HIV. Many of the early papers describing and applying causal inference methods had applications in either trial or observational data in this disease [3, 6, 49, 50], and as such it has had longer exposure to such methods, resulting in wider uptake. Therefore although it seems that researchers in specific disease areas are becoming aware of the need for causal methods, further efforts may be necessary to promote dissemination and uptake of such techniques into other therapeutic areas where they may be highly beneficial in gaining additional insights from existing trial data, rather than being exclusively used with observational data.

More broadly, a practical difficulty that may limit application of the methods in RCT settings may include the lack of power to estimate non-randomised effects. There is little methodological work examining power for causal analyses, and as such researchers may be unable to justify their use when developing analysis plans. In addition, lack of power in such analyses may often lead to inconclusive results, as is the case in many of the studies presented in this review. One exception to this was the study by Walker et al. [21]. In this case, the authors found strong evidence that cotrimoxazole use reduced mortality in the first 72 weeks after starting ART. However, overall, the lack of conclusive findings in many applications may result in scepticism of the benefits of causal methods, and in publication bias. As such, this review may actually underestimate how often causal methods are being applied in RCT settings, but without publication of results, the additional knowledge that may be gained from existing trials to generate hypotheses for subsequent clinical trials may be lost.

Strengths of the systematic review were its pre-defined protocol with a comprehensive search strategy. Further to this, the studies were reviewed against the pre-determined inclusion and exclusion criteria by four researchers, in order to minimise subjectivity. There is the possibility that a small number of relevant papers were missed due to restricting the search to English-language articles. However, despite this limitation, the final selection of papers is likely to provide a representative picture of the current use of causal methodology in RCTs beyond their use to adjust for compliance to randomised therapy or loss to follow up. Detailed data were taken from each paper to ensure good understanding of the motivation and methods used for each causal question examined and this enabled us to clearly describe how and where causal methods are being used within trials. The inclusion of more methodogical papers where an example application was given may slightly overestimate the use of the methods in some disease areas. For example, two cancer papers [32, 33] were mostly theoretical. However, their inclusion is still beneficial in terms of our aim of identifying areas in which causal methods are relevant and have potential.

By selecting studies that had used causal methodology to deal with issues of time-dependent confounding, it is likely that the methodology used was appropriate to answer the question of interest; however, we did not conduct any formal quality assessment of the included studies. For example, we did not examine whether the authors discussed (or conducted) sensitivity analyses to investigate whether or not necessary assumptions needed for valid causal inference were met. Although such quality assessment would have been important if we were attempting to use the studies to synthesise the evidence on a particular causal question, or to evaluate how rigorously such methodology is currently applied, for the main aims of this review we do not consider it to be a significant limitation. The main limitation is that our search was conducted in September 2014, which leaves the possibility that some more recent studies were missed. To assess this we conducted an updated search from January 2014 to September 2017 using the Web of Science database only, as this database provided 52% of the 2773 references found in the original search. After excluding references that were already in the original search to September 2014, there were 686 new records for screening. A brief screen of the first 250 articles (when ordered alphabetically by first author) identified 17 potentially relevant studies of which at further inspection, only one met the original inclusion criteria [47]. This equates to a 0.4% hit rate for this subsample of the updated search, compared to an overall rate of 0.9% in the original search, suggesting that the uptake of causal methodology in RCTs is unlikely to have substantially increased since the review was conducted.

## Conclusion

In conclusion, the use of causal methodology to answer additional questions from RCT data remains relatively limited. In particular, the use of the g-methods is minimal, potentially due to the more intuitive nature of IPW of MSMs making this the preferred approach for applied examples. The current applications and examples of causal methodology show that the methods can be implemented to answer questions on the use of concomitant medications, dosing strategies and treatment sequences and in some cases can provide clinically useful answers to questions not originally examined by the trial. It is possible that their use as a way to enhance current clinical trial data is under-emphasised due to an overall lack of clinically significant findings in the current literature. Further methodological work in terms of power calculations for causal methodology may be beneficial to enable trials to be designed to power secondary analyses, or at least make potential power issues more transparent. Further to this, there needs to be wider and more focussed efforts to make researchers more aware of causal methods, of how they can be implemented, and of their potential to add value to RCTs.

## Additional files

Additional file 1: Appendices 1 and 2. Contains systematic review search protocol, search terms and search logs from all databases. (DOCX 20 kb)

Additional file 2: Appendix 3. Full extraction table. Contains the full original extracted data from each article included in the review (XLSX 35 kb)

Additional file 3: PRISMA checklist. (DOCX 26 kb)

## Abbreviations

ALL: Acute lymphoblastic leukaemia
ART: Antiretroviral therapy
CD4: Cluster of differentiation 4
HIV: Human immunodeficiency virus
IPTW: Inverse probability of treatment weighting
IPW: Inverse probability weighting
ITT: Intention to treat
MSM: Marginal structural model
RCT: Randomised controlled trial
SMART: Sequential multiple assignment randomised trial
SNM: Structural nested model
