# Supplementary material for: Application of causal inference methods in the analyses of randomised controlled trials: a systematic review
Source: Trials. 2018 Jan 10;19:23. doi: 10.1186/s13063-017-2381-x (PMC5761133; doi:10.1186/s13063-017-2381-x)
Supplement: Supplementary file 1 — Contains systematic review search protocol, search terms and search logs from all databases. (DOCX 20 kb) [file 13063_2017_2381_MOESM1_ESM.docx]

**Additional file 1**

**Appendix 1: SEARCH PROTOCOL:**

| **Component** | **Description** |
| --- | --- |
| Review area | **Application of causal methods** |
| Objectives | To identify a) papers on the methodologies of causal methods and b) studies where these method have been used |
| Populations/aspect | All human/aspects include confounding, causality, epidemiological factors |
| Diagnosis | n/a |
| Interventions | Causal methods developed to address confounding such as marginal structural models, marginal nested models , inverse probability weighting, g-computation, g-estimation, g-formula |
| Comparisons/ aspects covered by search | No comparator |
| Study design | All study types |
| Exclusions | Animal studies/ economic evaluations /letters/editorials/anecdotes |
| How the information was searched | The results of two searches were combined with OR:  Search1:  Databases: Medline, Premedline, , Embase, Cochrane Library, Web of science  Language: English  date parameters: 1986-present  Search 2:  Prospective citation search of the 5 key papers listed below on Web of Science |
| Search terms and date searched | Search1:  (casual aspects) AND ( causal methods incl marginal structural models, marginal nested models , inverse probability weighting, g-computation, g-estimation, g-forrmula) LESS exclusions ( for details see Medline strategy attached)  searched 8/9/2014 |
| Search results | Medline/Premedline= 1092  Embase= 1081  Cochrane = 23 (1SR, 20 trials, 2 methods studies)  Web of science= 1017  Web of Science citations= 1407 unique citations ( retrieval from papers  1. 322 hits .2 128 hits 3. 934 hits 4. 240 hits 5. 396 hits)  Total = 4620  Total de-duplicated =2773 |
| Key papers | 1. Robins, JM. A New Approach to Causal Inference in Mortality Studies with a Sustained Exposure Period - Application to Control of the Healthy Worker Survivor Effect. Mathematical Modelling, (1986) (7) 9-12:1383-1512.  2. Robins JM. Association, causation, and marginal structural models. Synthese 1999; 121: 151–179.  3. Robins JM, Hernán MA, Brumback B. Marginal structural models and causal inference in epidemiology. Epidemiology 2000; 11(5): 550–560.  4. Cole SR, Hernán MA. Constructing inverse probability weights for marginal structural models. Am J Epidemiol 2008; 168(6): 656–664.  5. Hernán MA, Brumback B, Robins JM. Marginal structural models to estimate the causal effect of zidovudine on the survival of HIV-positive men. Epidemiology 2000; 11(5): 561–570. |

**Appendix 2: SEARCH STRATEGIES FROM EACH DATABASE**

**Medline**

Database: Medline In-process - Current week, Medline 1950 to present

Search Strategy:

--------------------------------------------------------------------------------

1 (causal inference and (method$ or approach$* or model$)).ti. (58)

2 "bias (epidemiology)"/ or causality/ or precipitating factors/ or "confounding factors (epidemiology)"/ or survival analysis/ or *epidemiologic methods/ or epidemiologic factors/ or exp effect modifier, epidemiologic/ (150382)

3 (causality or causal inference or causation or confounding$ or confounder$).ti,ab. (76503)

4 2 or 3 (219863)

5 models, statistical/ or logistic models/ or *models,theoretical/ or likelihood functions/ (219355)

6 (marginal structural or inverse probability or structural nested or counterfactual$ or semi-parametric or semiparametric or fully-parametric).ti,ab. (2928)

7 (causal adj3 (effect or effects or model$ or method$)).ti,ab. (4188)

8 5 and (6 or 7) (1678)

9 (g-computation or g-estimation or g-formula or doubly-robust estimation).ti,ab. (160)

10 (counterfactual or counterfactuals).ti,ab. (601)

11 (inverse probability adj3 (weight$ or estimat$)).ti,ab. (618)

12 ((marginal structural or structural nested or causal effect or causal effects or causal graphical or causal inference or semi-parametric or semiparametric or fully-parametric) adj3 (method$ or model$)).ti,ab. (1358)

13 or/8-12 (3328)

14 1 or (4 and 13) (1197)

15 *economics/ or exp *"costs and cost analysis"/ (57061)

16 (cost effectiv$ not clinical$).ti. (16830)

17 (health adj3 (economic or cost$)).ti. (5668)

18 letter/ (856890)

19 editorial/ (365454)

20 news/ (166586)

21 exp historical article/ (326805)

22 Anecdotes as topic/ (4607)

23 comment/ (602081)

24 case report/ (1711611)

25 (letter or comment$).ti. (99005)

26 or/18-25 (3412306)

27 randomized controlled trial/ or Randomized Controlled Trials as Topic/ or random$.ti,ab. (888123)

28 26 not 27 (3380930)

29 animals/ not humans/ (3913163)

30 exp Animals, Laboratory/ (738189)

31 exp Animal Experimentation/ (6483)

32 exp Models, Animal/ (426760)

33 exp rodentia/ (2715625)

34 (rat or rats or mouse or mice or rodent$).ti. (1148433)

35 or/28-34 (7910959)

36 15 or 16 or 17 or 35 (7974020)

37 14 not 36 (1116)

38 limit 37 to (english language and yr="1986 -Current") (1092)

**Cochrane:**

Search Name: causal methods

Date Run: 08/09/14 11:14:30.569

Description:

ID Search Hits

#1 (causal inference and (method* or approach* or model*)):ti 2

#2 MeSH descriptor: [Bias (Epidemiology)] this term only 607

#3 MeSH descriptor: [Causality] this term only 220

#4 MeSH descriptor: [Precipitating Factors] this term only 7

#5 MeSH descriptor: [Confounding Factors (Epidemiology)] this term only 368

#6 MeSH descriptor: [Epidemiologic Methods] this term only 903

#7 MeSH descriptor: [Epidemiologic Factors] this term only 8

#8 MeSH descriptor: [Effect Modifier, Epidemiologic] this term only 91

#9 (causality or "causal inference" or causation or confounding or confounder*):ti,ab,kw 4128

#10 #2 or #3 or #4 or #5 or #6 or #7 or #8 or #9 5682

#11 MeSH descriptor: [Models, Statistical] this term only 1328

#12 MeSH descriptor: [Logistic Models] this term only 4037

#13 MeSH descriptor: [Models, Theoretical] this term only 810

#14 MeSH descriptor: [Likelihood Functions] this term only 376

#15 ("marginal structural" or "structural nested" or counterfactual or "inverse probablity" or semi-parametric or semiparametric or fully-parametric):ti,ab,kw 90

#16 (causal near/3 (effect or effects or model* or method*)):ti,ab,kw 188

#17 (#11 or #12 or #13 or #14) and (#15 or #16) 27

#18 (g-computation or g-estimation or g-formula or "doubly-robust estimation" or counterfactual or counterfactuals):ti,ab,kw 34

#19 ((inverse-probablity or "inverse probablity") near/3 (weight* or estimat*)):ti,ab,kw 0

#20 (("marginal structural" or "structural nested" or "causal effect" or "causal effects" or "causal graphical" or "causal inference" or semi-parametric or semiparametric or fully-parametric) near/3 (model* or method*)):ti,ab,kw 57

#21 #17 or #18 or #19 or #20 102

#22 #1 or (#10 and #21) 23

#23 (cost effectiv* not clinical) .ti 20

#24 (health near/3 economic* or cost*) .ti 2495

#25 #23 or #24 2495

#26 #22 not #25 Publication Year from 1986 to 2014 23

**Embase**

Database: Embase <1974 to 2014 September 05>

Search Strategy:

--------------------------------------------------------------------------------

1 (causal inference and (method* or approach* or model*)).ti. (64)

2 *epidemiology/ or *confounding variable/ or *epidemiologic data/ or *causal attributes/ or *survival/ or *survival rate/ (67890)

3 (causality or causal inference or causation or confounding$ or confounder$).ti,ab. (94079)

4 2 or 3 (159289)

5 statistical model/ or *analytic method/ or statistical analysis/ or regression analysis/ or logistic regression analysis/ or maximum likelihood method/ or *theoretical model/ or causal modeling/ or "causal inference test"/ (474953)

6 (marginal structural or inverse probability or structural nested or semiparametric or semi-parametric or fully-parametric or counterfactual$).ti,ab. (3332)

7 (causal adj3 (effect or effects or model* or method*)).ti,ab. (4763)

8 5 and (6 or 7) (2315)

9 (g-computation or g-estimation or g-formula or doubly-robust estimation).ti,ab. (187)

10 (counterfactual or counterfactuals).ti,ab. (684)

11 (inverse probability adj3 (weight$ or estimat$)).ti,ab. (861)

12 ((marginal structural or structural nested or causal effect or causal effects or causal inference or causal graphical or semiparametric or semi-parametric or fully-parametric) adj3 (model$ or method$)).ti,ab. (1504)

13 or/8-12 (4216)

14 1 or (4 and 13) (1192)

15 letter.pt. or letter/ (858501)

16 note.pt. (568047)

17 editorial.pt. (455834)

18 case report/ or case study/ (1960646)

19 (letter or comment$).ti. (150815)

20 or/15-19 (3692007)

21 randomized controlled trial/ or "randomized controlled trial (topic)"/ or random$.ti,ab. (1022909)

22 20 not 21 (3655270)

23 exp animal/ not human/ (4422415)

24 nonhuman/ (4369325)

25 exp experimental animal/ (415618)

26 exp animal experiment/ (1801483)

27 exp animal model/ (785580)

28 exp rodent/ (2909106)

29 (rat or rats or mouse or mice or rodent*).ti. (1296812)

30 or/22-29 (10284619)

31 exp *health economics/ (196947)

32 *"cost benefit analysis"/ (7762)

33 (cost effectiv$ not clinical$).ti. (22511)

34 (health adj3 (economic or cost$)).ti. (6956)

35 30 or 31 or 32 or 33 or 34 (10461600)

36 14 not 35 (1108)

37 limit 36 to (english language and yr="1986 -Current") (1081)

***************************

**Web of science**

Run Search

Web of Science Core Collection

Search History - " Causal inf_veryfinal"

#14 #13

DocType=All document types; Language=All languages;

#13 (#11 not #12) AND LANGUAGE: (English)

DocType=All document types; Language=All languages;

#12 #10 OR #8 OR #7 OR #6

DocType=All document types; Language=All languages;

#11 #5 or #9

DocType=All document types; Language=All languages;

#10 TI=((cost-effectiv* not clinical*) or (health near/3 economic*) or ( health near/3 cost*))

DocType=All document types; Language=All languages;

#9 TI=(("causal inference" and method*) OR ("causal inference" and approach*) OR ("causal inference" and model*))

DocType=All document types; Language=All languages;

#8 TS=((animal* near/2 experiment*) or (animal* near/2 model*) or (mouse near/2 model*) or (animal* near/2 laborator*))

DocType=All document types; Language=All languages;

#7 TI= (letter* or anecdote* or editorial* or comment* or news)

DocType=All document types; Language=All languages;

#6 TI= ("mouse" or "mice" or "rat" or "rats" or rodent*)

DocType=All document types; Language=All languages;

#5 #4 AND #1

DocType=All document types; Language=All languages;

#4 #3 OR #2

DocType=All document types; Language=All languages;

#3 TS=(("marginal structural" near/3 model*) OR ("structural nested" near/3 model*) OR ("causal effect" near/3 model*) OR ( "causal effects" near/3 model*) or ("causal inference" near/3 model*) or ("causal graphical" near/3 model*) or (semiparametric near/3 model*) or (semi-parametric near/3 model*) or (fully-parametric near/3 model*) or ("causal effect" near/3 method*) OR ( "causal effects" near/3 method*) or ("causal inference" near/3 method*) or ("causal graphical" near/3 method*) or (semiparametric near/3 method*) or (semi-parametric near/3 method*) or (fully-parametric near/3 method*) or (" inverse probablity" near/3 weight*) or( "inverse probablity" near/3 etimat*))

DocType=All document types; Language=All languages;

#2 TS= (g-computation or g-estimation or g-formula or "doubly-robust estimation" or counterfactual or counterfactuals)

DocType=All document types; Language=All languages;

#1 TS= ((causality or "causal inference" or causation or confounding or confounder*) or (bias near/3 epidemiologic*) or ( survival near/3 rate*))

DocType=All document types; Language=All languages;
